# Radiographic outcomes following lateral alveolar ridge augmentation using autogenous tooth roots

**DOI:** 10.1186/s40729-018-0142-6

**Published:** 2018-09-28

**Authors:** Puria Parvini, Robert Sader, Didem Sahin, Jürgen Becker, Frank Schwarz

**Affiliations:** 1 Department of Oral Surgery and Implantology, Carolinum, Johann Wolfgang Goethe-University, Frankfurt, Germany; 2 Department for Oral, Cranio-Maxillofacial and Facial Plastic Surgery, Medical Center of the Goethe University Frankfurt, Frankfurt, Germany; 3 Department of Oral Surgery, Universitätsklinikum Düsseldorf, Düsseldorf, Germany

**Keywords:** Clinical study, Alveolar ridge augmentation, Tooth transplantation

## Abstract

**Background:**

To assess and compare the radiographic outcomes following lateral alveolar ridge augmentation using autogenous tooth roots (TR) and autogenous bone (AB) blocks.

**Methods:**

In a total of 30 patients, lateral ridge augmentation was conducted in parallel groups using either (1) healthy autogenous tooth roots (e.g., retained wisdom or impacted teeth) (*n* = 15) or (2) cortical autogenous bone blocks harvested from the retromolar area. Cone-beam computed tomographic (CBCT) scans taken at 26 weeks of submerged healing were analyzed for the basal graft integration (i.e., contact between the graft and the host bone in %) (BI26) and the cross-sectional grafted area (mm^2^) (SA26).

**Results:**

Both groups revealed a comparable clinical width of the alveolar ridge at baseline (CWb). Mean BI26 and SA26 values amounted to 69.26 ± 26.01% (median 72.44) and 22.07 ± 12.98 mm^2^ (median 18.83) in the TR group and 79.67 ± 15.66% (median 78.85) and 12.42 ± 10.11 mm^2^ (median 11.36) in the AB group, respectively. Between-group differences in mean SA26 values were statistically significant (*p* = 0.031). Linear regression analysis failed to reveal any significant correlations between BI26 and CWb/SA26 values in either group.

**Conclusions:**

TR grafts may be associated with improved SA26 values following lateral alveolar ridge augmentation.

**Trial registration:**

DRKS00009586. Registered 10 February 2016.

## Background

Autogenous bone (AB) blocks harvested from intraoral donor sites (i.e., retromandibular, chin) are the most commonly used procedure for lateral alveolar ridge augmentation [1]. However, despite significant horizontal bone gains, cortical bone blocks were noted to undergo an incomplete replacement resorption [2, 3], thus featuring a composition of non-vital residual and newly formed vital bone in the former defect area [4]. Moreover, AB blocks are prone to a rapid degradation and therefore commonly combined with contour augmentation procedures using slowly resorbing particulate grafts and barrier membranes [5].

Recent experimental studies have focused on the use of extracted tooth roots (TR) as an alternative scaffold to support bone regeneration at non-self-contained lateral alveolar ridge defects. Various outcome measures based on histological, immunohistochemical, and micro-computed tomographic analyses did not significantly differ between differently conditioned TRs (i.e., healthy, endodontically treated non-infected, periodontally diseased) and retromolar AB grafts [4, 6, 7]. The median bone-to-implant contact (BIC) values at 3 weeks following implant placement ranged from 36.96 to 50.79% in the TR group and from 32.53 to 64.10% in the AB group [4].

These preclinical data have recently been in an initial human case report [8] as well as in a prospective controlled clinical study [9]. In particular, soft tissue healing was uneventful in both TR and AB groups. The crestal ridge width at 26 weeks (CW26) amounted to 10.06 ± 1.85 mm (median 11.0) in the TR group and 9.20 ± 2.09 mm (median 8.50) in the AB group and allowed for a successful implant placement in all patients investigated [9].

The aim of the present analysis was to assess and compare the radiographic outcomes in both groups.

## Methods

### Study design and participants

This analysis was based on the radiographic (i.e., cone-beam computed tomographic—CBCT) data derived from a prospective controlled clinical monocenter study including a total of 30 patients [9]. Each participant exhibited either a tooth gap or a free-end situation with an inadequate horizontal ridge width and was in need of an implant-supported fixed restoration.

In brief, lateral ridge augmentation was conducted according to a standardized procedure under local anesthesia [8].

One group of patients (*n* = 15; mean age 41.93 years; range 19 to 60 years) exhibited either one or more caries-free partially/fully retained or impacted wisdom teeth without signs of local pathologies (e.g., cysts). TR grafts were separated (i.e., crown decapitation, vertical separation of multi-rooted teeth, preservation of the exposed pulp) from the extracted/surgically removed teeth and adapted in size and shape to match the defect area. At the respective downward aspect of the TR graft, the layer of cementum was carefully removed using a diamond bur to facilitate ankylosis at the recipient site [4].

Due to the absence of any suitable wisdom teeth, another group of patients (*n* = 15; mean age 44.53 years; range 21 to 60 years) was allocated to the harvesting of monocortical block grafts from the linea obliqua. Both TR and AB grafts were rigidly fixed using one to two titanium osteosynthesis screws (1.5 × 9 mm, Medicon, Tuttlingen, Germany) after gently flattening the recipient site using a round carbide bur underwater (i.e., sterile saline) cooling.

**Fig. 1:**
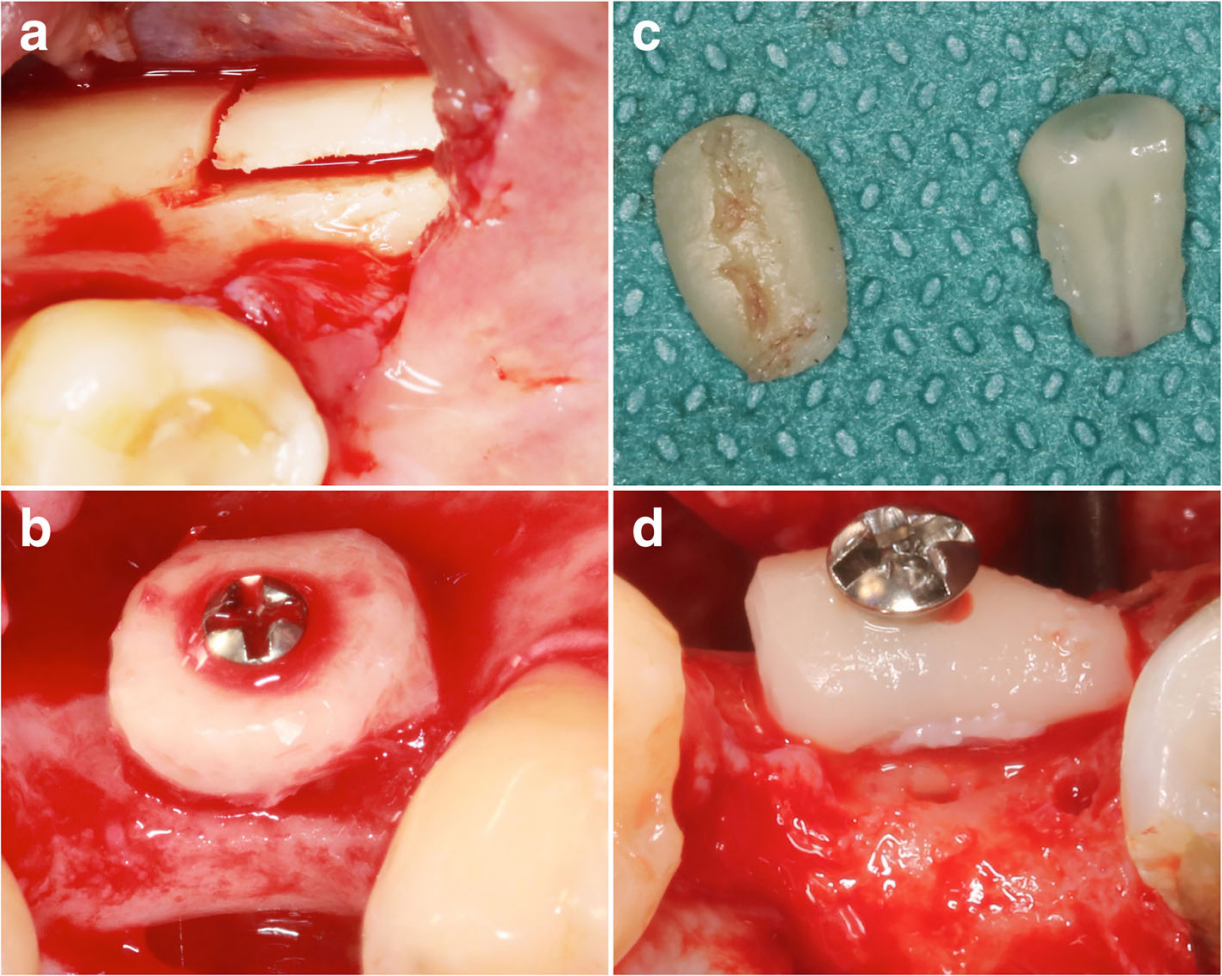
Lateral ridge augmentation—a surgical procedure in the AB and TR groups. **a** The retromolar area served as a donor site for the harvesting of monocortical bone blocks in the AB group. **b** AB blocks were shaped to match the size and configuration of the defect site and fixed using one central osteosynthesis screw. **c** TR grafts were separated from either partially/fully retained or impacted wisdom teeth. **d** The most suitable specimen was positioned and fixed in a way that the exposed dentin faced the defect area, thus facilitating ankylosis at the recipient site. The crestal perforations were derived from initial attempts to pre-drill the osteosynthesis screw. All sites were left to heal in a submerged position without providing any contour augmentation procedures

Advancement of the mucoperiosteal flaps was achieved using periosteal-releasing incisions. The coronally repositioned flaps were fixed using vertical double sutures to allow for a submerged healing period of 26 weeks (Fig. 1).

All patients had received a perioperative antibiotic (1× amoxicillin 2 g) as well as a peri- and postoperative (2 days) antiphlogistic prophylaxis (prednisolon, total of 40 mg). Analgetics (ibuprofen 600 mg) were provided according to individual needs.

The study outline and the follow-up visits are summarized in Table 1 [9].

**Table 1:** Study design and follow up visits

Visit 1	Visit 2	Visit 3	Visit 4	Visit 5	Visit 6
Recruitment	Surgery				Re-entry
	D0	D10	W4	W13	W26

*D* day, *W* week

### Ethics, consent, and permissions

Each patient was given a detailed description of the study procedures and signed a consent to participate. The study protocol was approved by the ethics committee (4837R) of the Heinrich Heine University, Düsseldorf, Germany, and registered via the Internet Portal of the German Clinical Trials Register (DRKS00009586).

The present reporting considered the checklist items as proposed in the STROBE statement.

### Inclusion and exclusion criteria

The inclusion criteria considered the following conditions: (1) age 18 to 60 years; (2) candidate for lateral ridge augmentation; (3) insufficient bone ridge width associated with a non-contained defect at the recipient site for implant placement, as evidenced intraoperatively; (4) sufficient bone height at the recipient site for implant placement, as evidenced in a preoperative panoramic radiograph; and (5) healthy oral mucosa, at least 3 mm keratinized tissue.

The exclusion criteria included the following conditions: (1) general contraindications for dental and/or surgical treatments; (2) inflammatory and autoimmune disease of the oral cavity; (3) uncontrolled diabetes (HbA1c > 7%); (4) history of malignancy requiring chemotherapy or radiotherapy within the past 5 years; (5) previous immunosuppressant, bisphosphonate, or high-dose corticosteroid therapy; (6) smokers; and (7) pregnant or lactating women [9].

### Clinical assessments

The clinical width (CW) of the alveolar ridge immediately before the augmentation (CWb) was assessed to the nearest 0.25 mm by means of a caliper. This was positioned at 2 mm below the crest at the most central aspect of the respective defect site, whose vertical plane was marked by the osteosynthesis screw. Measurement of CW was repeated immediately after augmentation (CWa). Graft thickness (GT) was calculated as CWa − CWb.

### Radiographic assessments

According to the clinical standard procedure, CBCT scans (25 patients: PaX-i3D Green, Orangedental, Biberach, Germany, at 95 kV, 8.5–9.0 mAs; 5 patients: ProMax3D, Planmeca, Helsinki, Finland, at 90 kV, 5.6–9.0 mAs) using adjusted fields of view (i.e., 5 × 5 and 8 × 5 cm) were taken at 26 weeks for preoperative implant planning at the respective sites.

**Fig. 2:**
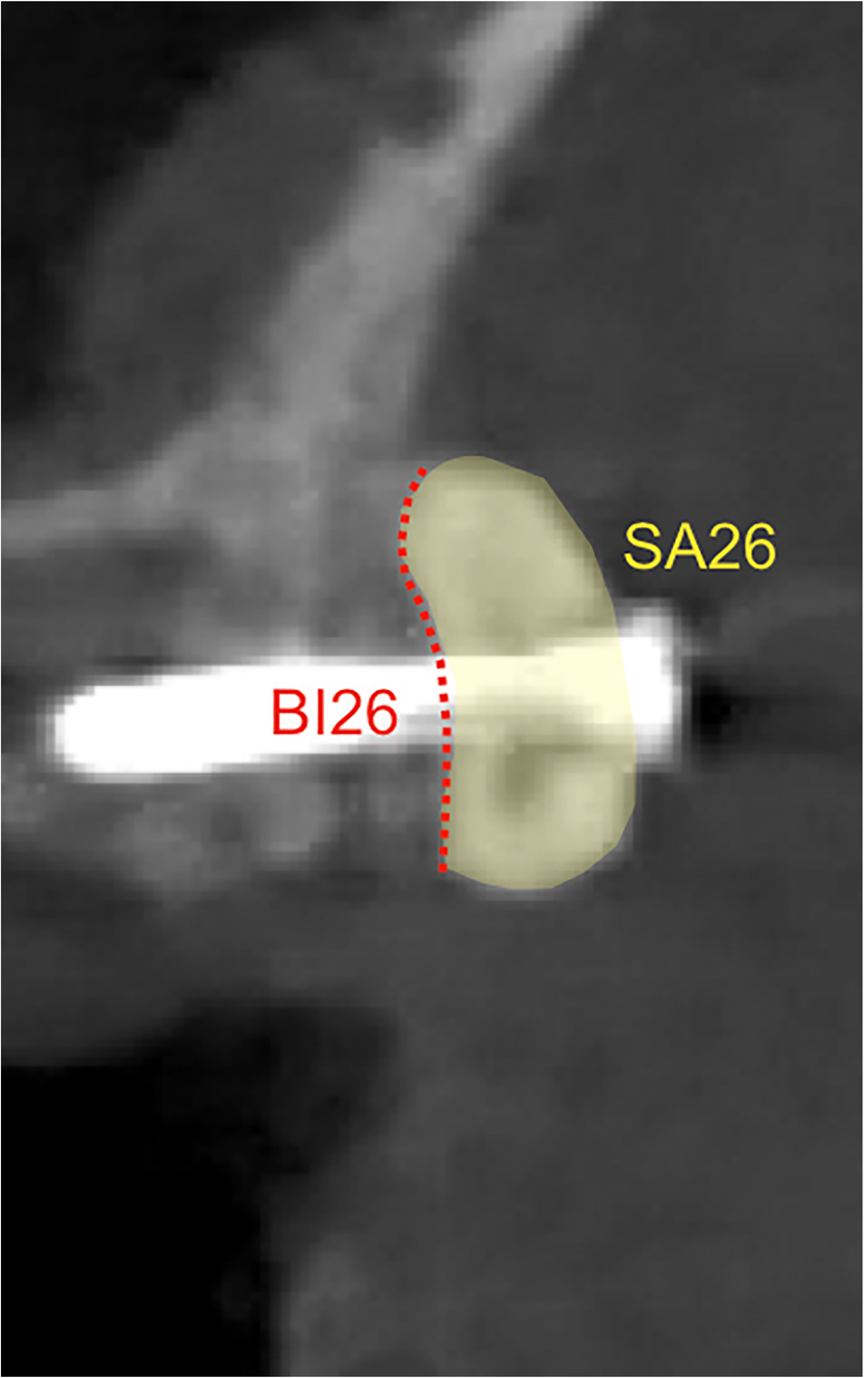
Radiographic assessments. Images of the coronal planes representing the most central aspect of the respective defect sites were analyzed for the basal graft integration (i.e., contact between the graft and the host bone in %) (BI26) and the cross-sectional grafted area (mm^2^) (SA26)

Images of the coronal planes representing the most central aspect of the respective defect sites were exported and analyzed for the basal graft integration (BI26) and the cross-sectional grafted area (mm^2^) (SA26) (ImageJ). In particular, BI26 was measured as a percentage AB/TR to host bone contact along the basal graft extension serving as 100%, respectively (Fig. 2).

All measurements were performed by one previously calibrated investigator.

### Sample size calculation and statistical analysis

The sample size calculation considered a standard normal distribution (type I error set at .05; type II error set at .20) and a sigma which was estimated based on the standard deviations observed in a recent preclinical animal study [4]. The clinical width of the alveolar ridge was defined as the primary outcome variable, considering a clinically relevant difference of 2 mm. A sample size of 15 patients per group was calculated to achieve a 95% power (Power and Precision, Biostat, Englewood, USA).

The statistical analysis of the pseudonymized data sets was accomplished using a commercially available software program (IBM SPSS Statistics 24.0, IBM Corp., Armonk, NY, USA).

Mean values, standard deviations, medians, 95% confidence intervals (CI), and frequency distributions were calculated for all outcomes assessed. The data rows were examined with the Shapiro-Wilk test for normal distribution. Between-group comparisons were accomplished using the unpaired *t* test. Linear regression analyses were used to depict the relationship between BI26 and CWb as well as SA26 values in both groups. The alpha error was set at 0.05.

## Results

Mean CWb and GT values were comparable in both groups and amounted to 4.53 ± 1.54 mm (median 4.50; 95% CI 3.68, 5.38) and 5.66 ± 1.75 mm (median 5.0; 95% CI 4.69, 6.64) in the TR group and 5.26 ± 1.25 mm (median 5.00; 95% CI 4.57, 5.95) and 4.96 ± 1.75 mm (median 5.0; 95% CI 4.24, 5.68) in the AB group, respectively. Between-group differences did not reach statistical significance.

### Radiographic performance endpoints

Mean SA26 values were 12.42 ± 10.11 mm^2^ (median 11.36; 95% CI 6.82, 18.02) in the AB group and amounted to 22.07 ± 12.98 mm^2^ (median 18.83; 95% CI 14.88, 29.26) at the TR-treated sites. The resulting differences between both groups were statistically significant (*p* = 0.031).

**Fig. 3:**
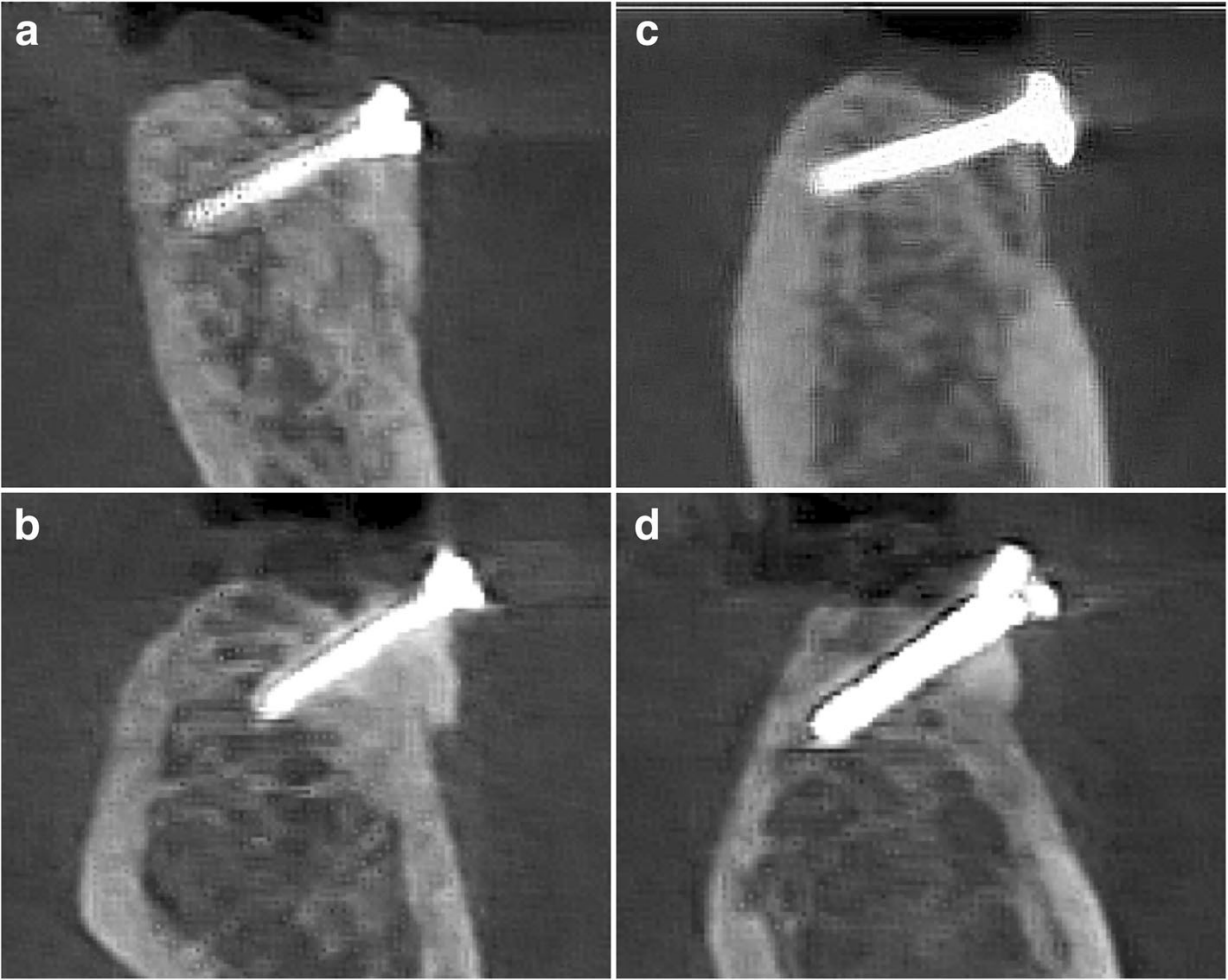
Representative CBCT outcomes at 26 weeks. **a**, **b** TR graft. **c**, **d** AB graft

Mean BI26 values amounted to 79.67 ± 15.66% (median 78.85; 95% CI 70.99, 88.34) in the AB group and tended to be lower at the TR-treated sites, revealing a mean value of 69.26 ± 26.01% (median 72.44; 95% CI 53.85, 82.66) (Fig. 3). These differences, however, failed to reach statistical significance (*p* = 0.157) (Table 2).

**Table 2:** Secondary performance endpoints (in mm)

	CWb	GT	SA26	BI26
a) TR group (*n* = 15 patients)				
Mean	4.53	5.66	22.07*	69.26
SD	1.54	1.75	12.98	26.01
Median	4.50	5.00	18.83	72.44
95% CI	3.68, 5.38	4.69, 6.64	14.88, 29.26	53.85, 82.66
b) AB group (*n* = 15 patients)				
Mean	5.26	4.96	12.42	79.67
SD	1.25	1.30	10.11	15.66
Median	5.00	5.00	11.36	78.85
95% CI	4.57, 5.95	4.24, 5.68	6.82, 18.02	70.99, 88.34

Comparisons between the groups (unpaired *t* test): *p = 0.031

*CWb* clinical width of the alveolar ridge immediately before augmentation (D0) (mm), *GT* graft thickness immediately after augmentation (D0) (mm), *SA26* surface area at 26 weeks (W26) (mm^2^), *BI26* basal integration at 26 weeks (W26) (%)

### Regression analysis

**Fig. 4:**
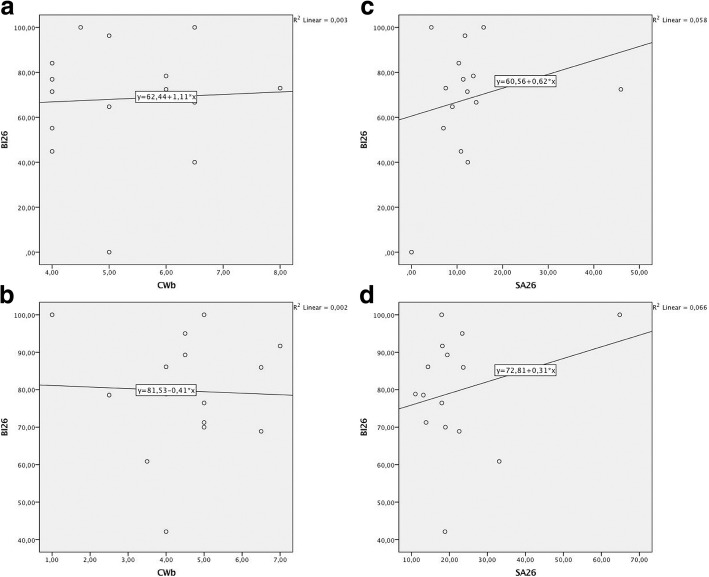
Linear regression plots to depict the relationship between BI26 and CWb/SA26 values. **a** CWb (TR group). **b** CWb (AB group). **c** SA26 (TR group). **d** SA26 (AB group)

In both groups investigated, the linear regression analysis failed to reveal any significant correlations between BI26 and CWb (TR: Coef. 1.106; *R*^2^ = 0.003; *p* = 0.851; AB: Coef. − 0.410; *R*^2^ = 0.002; *p* = 0.886) or BI26 and SA26 (TR: Coef. 0.619; *R*^2^ = 0.058; *p* = 0.387; AB: Coef. 0.311; *R*^2^ = 0.066; *p* = 0.354) values, respectively (Fig. 4).

## Discussion

The present analysis aimed at assessing and comparing CBCT outcomes following lateral alveolar ridge augmentation using TR and AB grafts. After a healing period of 26 weeks, it was observed that TR grafts were associated with significantly higher mean SA26 values when compared with the AB group. A similar tendency was also noted for mean BI26 values; however, this difference did not reach statistical significance.

When interpreting these results, it must be kept in mind that both groups were associated with comparable CWb and GT values at baseline. However, the clinical re-entry at 26 weeks revealed that mean CW values amounted to 10.06 ± 1.85 mm (median 11.0; 95% CI 9.03, 11.09) in the TR group and 9.20 ± 2.09 mm (median 8.50; 95% CI 8.04, 10.35) in the AB group, respectively. This was associated with a significantly higher gain in ridge width of 5.53 ± 1.88 mm (median 5.00; 95% CI 4.48, 6.57) at TR- over the AB-treated sites (3.93 ± 1.41 mm; median 4.00; 95% CI 3.15, 4.71) [9]. This difference was mainly due to a lower graft resorption in the TR group, which was basically confirmed by the present analysis of SA26 values. Moreover, a recent animal study employing both TR and AB grafts for lateral alveolar ridge augmentation also corroborates, at least in part, the differences in mean SA26 values noted between both groups. In particular, after 12 weeks of healing, the histomorphometrical analysis of the augmented area (AA) at the TR-treated sites ranged between 7.55 and 11.20 mm^2^, whereas the median values ranged between 6.60 and 8.56 mm^2^ at the AB-treated sites [4]. Similar AA values were also noted when assessing the efficacy of TR grafts that were derived from the periodontally diseased teeth, resulting in 11.01 ± 4.37 mm^2^ as compared to 8.07 ± 5.64 mm^2^ noted in the AB group [6].

However, previous clinical studies suggest that the resorption of AB grafts may be limited by a simultaneous contour augmentation (e.g., application of a bovine-derived xenograft and coverage by a native collagen membrane) [5, 10]. In particular, CBCT analyses at 10 years revealed only a minor superficial resorption of about 7.7%, which corresponded to 0.38 mm [10].

When further analyzing the present data, it was also noted that both TR and AB grafts were associated with comparable BI26 values, thus corroborating the clinical observation of a firm graft connection to the host bone at 26 weeks, which allowed for a proper placement of adequately dimensioned titanium implants at all sites investigated [9]. The regression analysis also revealed that BI26 values were neither related to CWb nor SA26 values. These clinical and radiographic observations are also supported by recent histological analyses pointing to a basal ankylosis and replacement resorption of both TR and AB grafts [4, 6, 7].

## Conclusions

In conclusion and within its limitations, the present clinical study revealed that TR grafts may be associated with improved SA26 values following lateral alveolar ridge augmentation.
